# New Molecular Tools for Regulation and Improvement of A40926 Glycopeptide Antibiotic Production in *Nonomuraea gerenzanensis* ATCC 39727

**DOI:** 10.3389/fmicb.2020.00008

**Published:** 2020-01-21

**Authors:** Oleksandr Yushchuk, Andres Andreo-Vidal, Giorgia Letizia Marcone, Mervyn Bibb, Flavia Marinelli, Elisa Binda

**Affiliations:** ^1^Department of Biotechnology and Life Sciences, University of Insubria, Varese, Italy; ^2^Department of Molecular Microbiology, John Innes Centre, Norwich Research Park, Norwich, United Kingdom

**Keywords:** A40926, *Nonomuraea*, glycopeptide antibiotics, pathway-specific regulators, strain improvement

## Abstract

Genome sequencing has revealed that *Nonomuraea* spp. represent a still largely unexplored source of specialized metabolites. *Nonomuraea gerenzanensis* ATCC 39727 is the most studied representative species since it produces the glycopeptide antibiotic (GPA) A40926 – the precursor of the clinically relevant antibiotic dalbavancin, approved by the FDA in 2014 for the treatment of acute skin infections caused by multi-drug resistant Gram-positive pathogens. The clinical relevance of dalbavancin has prompted increased attention on A40926 biosynthesis and its regulation. In this paper, we investigated how to enhance the genetic toolkit for members of the *Nonomuraea* genus, which have proved quite recalcitrant to genetic manipulation. By constructing promoter-probe vectors, we tested the activity of 11 promoters (heterologous and native) using the GusA reporter system in *N. gerenzanensis* and in *Nonomuraea coxensis*; this latter species is phylogenetically distant from *N. gerenzanesis* and also possesses the genetic potential to produce A40926 or a very similar GPA. Finally, the strongest constitutive promoter analyzed in this study, *aac(3)IVp*, was used to overexpress the cluster-situated regulatory genes controlling A40926 biosynthesis (*dbv3* and *dbv4* from *N. gerenzanensis* and *nocRI* from *N. coxensis*) in *N*. *gerenzanensis*, and the growth and productivity of the best performing strains were assessed at bioreactor scale using an industrial production medium. Overexpression of positive pathway-specific regulatory genes resulted in a significant increase in the level of A40926 production in *N. gerenzanensis*, providing a new knowledge-based approach to strain improvement for this valuable glycopeptide antibiotic.

## Introduction

Research on glycopeptide antibiotics (GPAs) – drugs of “last resort” for treating severe infections caused by multi-drug resistant Gram-positive pathogens – has experienced a “renaissance” over the last decade (Marcone et al., 2018). Clinically important GPAs include two natural products (vancomycin and teicoplanin) and three second generation antibiotics (telavancin, dalbavancin, and oritavancin), which are semisynthetic derivatives of natural products endowed with an increased antimicrobial potency and superior pharmacokinetic properties. The urgent need for new potent antibiotics has driven much recent interest in GPAs. For example, chemical synthesis recently resulted in the generation of a plethora of vancomycin derivatives with novel modifications that show superior antimicrobial activities (Okano et al., 2017; Wu and Boger, 2019), while teicoplanin has been conjugated with nanoparticles resulting in increased activity against biofilm-forming pathogens (Armenia et al., 2018).

In contrast, genetic manipulation of GPA producers to yield novel potent derivatives is in its infancy. Recent work (Haslinger et al., 2015; Peschke et al., 2017; Schoppet et al., 2019) has revealed new details of the specificity and timing of non-ribosomal peptide synthesis, including chlorination and cross-linking steps, suggesting that the use of combinatorial biosynthesis to generate GPAs with completely novel oligopeptide scaffolds should be possible. In parallel, heterologous expression of enzymes involved in later stages of GPA biosynthesis (glycosylation, sulfation, acylation etc.) in known producers or *in vitro* has already generated novel GPA derivatives that could not be prepared easily by chemical synthesis (Banik and Brady, 2008; Banik et al., 2010; Yim et al., 2014).

In the meantime, genome sequencing has revealed the organization of GPA biosynthetic gene clusters (BGCs) in industrially valuable actinobacteria (D’Argenio et al., 2016), including long known GPA-producers (Vongsangnak et al., 2012; Truman et al., 2014; Kusserow and Gulder, 2017; Nazari et al., 2017; Adamek et al., 2018) as well as in novel producing strains (Thaker et al., 2013; Stegmann et al., 2014). Although the global regulation of GPA biosynthesis is still largely unexplored, the pathway-specific regulation controlling the expression of BGCs is being elucidated in model systems (Bibb, 2013). The roles of cluster-situated regulatory genes have been investigated in *Amycolatopsis balhimycina* (Shawky et al., 2007), *Nonomuraea gerenzanensis* (Lo Grasso et al., 2015; Alduina et al., 2018), and *Actinoplanes teichomyceticus* (Horbal et al., 2014b; Yushchuk et al., 2019), producing balhimycin, A40926 (the natural precursor of dalbavancin), and teicoplanin, respectively. Overexpression of the teicoplanin cluster-situated regulatory genes (tei15*, coding for a StrR-like transcriptional regulator, and tei16*, coding for a LuxR*-*type regulator) in *A. teichomyceticus* markedly increased teicoplanin production in the wild type strain, representing one of the most successful examples of using molecular tools for improving antibiotic production (Horbal et al., 2012, 2014b). In this work, we investigated the potential of molecular tools to improve the production of A40926, the dalbavancin precursor.

The BGC for A40926, named *dbv*, contains two genes encoding transcriptional regulators: *dbv3* and *dbv4* (Lo Grasso et al., 2015). *dbv3* encodes a LuxR-type regulator, which, however, is non-orthologous to the *tei* cluster encoded LuxR-regulator – Tei16* (Yushchuk et al., 2019). *dbv4* codes for a StrR-like transcriptional regulator with close homologues in every GPA BGC (Yushchuk et al., 2019). The A40926-producing strain, recently re-classified as *N. gerenzanensis* (Dalmastri et al., 2016), belongs to a still poorly investigated genus of actinobacteria that was only recently identified as an untapped source of novel antibiotics and other bioactive metabolites (Sungthong and Nakaew, 2015). More recently, fully sequenced genomes of *N. gerenzanensis* (D’Argenio et al., 2016) and of the kistamicin producer *Nonomuraea* sp. ATCC 55076, previously classified as *Actinomadura parvosata* subsp. *kistnae* S382–8 (Kusserow and Gulder, 2017; Nazari et al., 2017), have confirmed the hidden potential of these uncommon actinomycetes as prolific producers of specialized metabolites. In this paper, we report that another member of this genus, *Nonomuraea coxensis* DSM 45129, which was isolated in Bangladesh in 2007 (Ara et al., 2007), has the genetic potential to produce A40926 or a very similar GPA; its BGC contains two regulatory genes, *nocRI* and *nocRII*, which are close homologs of *dbv3* and *dbv4*, respectively. Thus, we first developed the molecular tools to manipulate both *N. gerenzanensis* and *N. coxensis*, we then selected the strongest heterologous promoter to drive gene expression in *Nonomuraea* spp., and finally we overexpressed both native and heterologous cluster-specific regulatory genes in *N. gerenzanensis*, assessing the best performers at flask and bioreactor scale in industrial media. The overexpression of the positive pathway-specific regulators significantly increased the level of A40926 production in *N. gerenzanensis*, paving the way for knowledge-based strain improvement for the production of this valuable GPA.

## Materials and Methods

### Plasmids, Bacterial Strains, Antibiotics, and Culture Conditions

Plasmids and bacterial strains used in this work are summarized in Table 1. Compositions of media are given in ESM. Unless otherwise stated, all media components and antibiotics were supplied by Sigma-Aldrich, St. Louis, MO, United States. For routine maintenance, actinobacterial strains were cultivated on ISP3 or VM0.1 agar media supplemented with 50 μg/ml apramycin-sulfate when appropriate. For genomic DNA isolation, *N. gerenzanensis* ATCC 39727 and *N. coxensis* DSM 45129 were grown in 250 ml Erlenmeyer flasks containing 10 glass beads (ø5 mm) with 50 ml of liquid VSP medium on an orbital shaker at 220 rpm and at 30°C. Working cell banks (WCB) of *Nonomuraea* spp. were prepared as described previously (Marcone et al., 2014). *Escherichia coli* DH5α was used as a routine cloning host and *E. coli* ET12567 pUZ8002 as a donor for intergeneric conjugations. *E. coli* strains were grown at 37°C in LB liquid or agar media supplemented with 100 μg/ml of apramycin-sulfate, 50 μg/ml of kanamycin-sulfate and 25 μg/ml of chloramphenicol when appropriate.

**Table 1:** Bacterial strains and plasmids used in this work.

Name	Description	Source of reference
*N. gerenzanensis*	Wild type, A40926 producer	ATCC 39727
*N. coxensis*	Wild type	DSM 45129
*N. gerenzanensis* pSET152A^+^	Wild type derivative carrying pSET152A	This work
*N. gerenzanensis* pSAD3^+^	Wild type derivative carrying pSAD3	This work
*N. gerenzanensis* pSAD4^+^	Wild type derivative carrying pSAD4	This work
*N. gerenzanensis* pSAD3–4^+^	Wild type derivative carrying pSAD3–4	This work
*N. gerenzanensis* pSAR1^+^	Wild type derivative carrying pSAR1	This work
*N. gerenzanensis* pSAGA^+^	Wild type derivative carrying pSAGA	This work
*N. gerenzanensis* pTEGA^+^	Wild type derivative carrying pTEGA	This work
*N. gerenzanensis* pGUSmoeE5script^+^	Wild type derivative carrying pGUSmoeE5script	This work
*N. gerenzanensis* pGCymRP21^+^	Wild type derivative carrying pGCymRP21	This work
*N. gerenzanensis* pGT2p^+^	Wild type derivative carrying pGT2p	This work
*N. gerenzanensis* pGBP^+^	Wild type derivative carrying pGBP	This work
*N. gerenzanensis* pHBP^+^	Wild type derivative carrying pHBP	This work
*N. gerenzanensis* pSBP^+^	Wild type derivative carrying pSBP	This work
*N. gerenzanensis* pRLP^+^	Wild type derivative carrying pRLP	This work
*N. gerenzanensis* pRBP1^+^	Wild type derivative carrying pRBP1	This work
*N. gerenzanensis* pRBP2^+^	Wild type derivative carrying pRBP2	This work
*N. coxensis* pSAGA^+^	Wild type derivative carrying pSAGA	This work
*N. coxensis* pTEGA^+^	Wild type derivative carrying pTEGA	This work
*N. coxensis* pGUSmoeE5script^+^	Wild type derivative carrying pGUSmoeE5script	This work
*N. coxensis* pGCymRP21^+^	Wild type derivative carrying pGCymRP21	This work
*N. coxensis* pGT2p^+^	Wild type derivative carrying pGT2p	This work
*N. coxensis* pGBP^+^	Wild type derivative carrying pGBP	This work
*N. coxensis* pHBP^+^	Wild type derivative carrying pHBP	This work
*N. coxensis* pSBP^+^	Wild type derivative carrying pSBP	This work
*N. coxensis* pRLP^+^	Wild type derivative carrying pRLP	This work
*N. coxensis* pRBP1^+^	Wild type derivative carrying pRBP1	This work
*N. coxensis* pRBP2^+^	Wild type derivative carrying pRBP2	This work
*E. coli* DH5α	General cloning host	MBI Fermentas, USA
*E. coli* ET12567 pUZ8002	(*dam-13*::Tn9 *dcm-6*), pUZ8002^+^ (Δ*oriT*), used for conjugative transfer of DNA	Kieser et al. (2000)
A40Y	SuperCos1 derivative, including 22 kb of *dbv* cluster (*dbv1-dbv17*)	Marcone et al. (2010a)
pGUS	pSET152 derivative, containing promoterless *gusA*	Myronovskyi et al. (2011)
pSET152A	pSET152 derivative, containing *aac(3)IVp* from pIJ773	Horbal et al. (2013)
pSAD3	pSET152A derivative, containing *dbv3* under the control of *aac(3)IVp*	This work
pSAD4	pSET152A derivative, containing *dbv4* under the control of *aac(3)IVp*	This work
pSAD3–4	pSET152A derivative, containing *dbv4* together with *dbv3*	This work
pSAR1	pSET152A derivative, containing *nocRI* under the control of *aac(3)IVp*	This work
pSAGA	pSET152A derivative, containing *gusA* under the control of *aac(3)IVp*	Koshla et al. (2019)
pTEGA	pTES derivative, containing *gusA* under the control of *ermEp*	Yushchuk et al. (2020a)
pGUSmoeE5script	pGUS derivative, containing *gusA* under the control of *moeE5p*	Makitrynskyy et al. (2013)
pGCymRP21	pGUS derivative, containing CymR operator, *P21* promoter and *cymR* gene	Horbal et al. (2014a)
pGT2p	pGUS derivative, containing *gusA* under the control of *tei2p*	Yushchuk et al. (2020b)
pGBP	pSAGA derivative, containing *gusA* under the control of *gyrB_ng_p*	This work
pHBP	pSAGA derivative, containing *gusA* under the control of *hrdB_ng_p*	This work
pSBP	pSAGA derivative, containing *gusA* under the control of *ssb_ng_p*	This work
pRLP	pSAGA derivative, containing *gusA* under the control of *rpsL_ng_p*	This work
pRBP1	pSAGA derivative, containing *gusA* under the control of *rpoB_ng_p*	This work
pRBP2	pSAGA derivative, containing *gusA* under the control of *rpoB_Rng_p*	This work

### Generation of Recombinant Plasmids

#### Construction of Promoter-Probe Vectors

To test the activity of different native *N. gerenzanensis* promoters, pSAGA (Koshla et al., 2019), where *gusA* (Myronovskyi et al., 2011) is expressed from *aac(3)IVp*, was chosen as a chassis. Genomic DNA, extracted from *N. gerenzanensis* according to the Kirby procedure (Kieser et al., 2000), was used as a template to amplify the putative promoter regions of: *BN4615_P641* (coding for the DNA gyrase B subunit – GyrB_ng_) 342 bp; *BN4615_P8899* (coding for the RNA polymerase sigma factor RpoD – HrdB_ng_), 524 bp; *BN4615_P604* (coding for the single-stranded DNA-binding protein – Ssb_ng_), 207 bp; *BN4615_P1543* (coding for the SSU ribosomal protein S12p – RpsL_ng_), 339 bp; *BN4615_P7269* (coding for the rifamycin-resistant RNA polymerase subunit β – RpoB_Rng_), 558 bp; and *BN4615_P1539* (coding for the rifamycin-sensitive RNA polymerase subunit β – RpoB_ng_), 493 bp. Amplicons were generated using Q5 High-Fidelity DNA Polymerase (New England Biolabs, Ipswich, MA, United States) according to the supplier’s protocol and the oligonucleotide primers listed in Table 2. All of the amplicons were digested with *Bam*HI and *Eco*RV and cloned in pSAGA cleaved with the same endonucleases, thus replacing *aac(3)IVp* in front of *gusA* with each of the amplified promoter regions. The resulting recombinant plasmids were named pGBP (carrying *gyrB_ng_p*), pHBP (*hrdB_ng_p*), pSBP (*ssb_ng_p*), pRLP (*rpsL_ng_p*), pRBP1 (*rpoB_ng_p*), and pRBP2 (*rpoB_Rng_p*).

**Table 2:** Oligonucleotide primers used in this work.

Primer	Nucleotide sequence (5′-3′)^*^	Purpose
Dbv3_F Dbv3_R	TTTGATATCGGAGGGCAAGAGTGCTGTTCGGGC TTTGAATTCCTCTACAGCCGCACTGCCT	Cloning of *dbv3* into pSET152A
dbv4_F dbv4_R	TTTGATATCGGAGGGGCTAGGTGGACCCGACGG TTTGAATTCTCCACTCGTGCTCATCCAG	Cloning of *dbv4* into pSET152A
PAM_seq_F dbv3_seq_R orfR1nid_EcoRV_rev	GATGTCATCAGCGGTGGAG CCAGCGCTGGACCGCCTGC TTTGATATCGCAAGGGGCCTCCCCGCCG	Verification of recombinant strains
orfR1_F orfR1_R	TTTGATATCGGAGGACTGCGTTGACGAACCGCT TTTGAATTCGCGTCATGGGACCACCGCC	Cloning of *nocRI* into pSET152A
hrdBp_F hrdBp_R	TTTGGATCCACCGAAGCGCCGCCTGAGG TTTGATATCGAAGGCCTGACGGACATCC	Cloning of *hrdB_ng_p*
rpoB1p_F rpoB1p_R	TTTGGATCCTCTCGCTGGCTGGTGGCCG TTTGATATCTCGCGGCGGACTGACTACA	Cloning of *rpoB_ng_p*
rpoB2p_F rpoB2p_R	TTTGGATCCTGTCGTACTGCTC TTTGATATCATACGAAGGCGAGGGAGGG	Cloning of *rpoB_Rng_p*
rpsLp_F rpsLp_R	TTTGGATCCATGGACGGCGGAGCTGTAG TTTGATATCTTGGCCGGTGTTACGTCA	Cloning of *rpsL_ng_p*
ssbp_F ssbp_R	TTTGGATCCAAGTCCGAAGGCATCTACG TTTGATATCTGCACGCCTTCGCTTGGGT	Cloning of *ssb_ng_p*
gyrBAp_F gyrBAp_R	TTTGGATCCAGGCTTCGCACAGTAACGG TTTGATATCGCGGACACGCGGCGGGGGA	Cloning of *gyrB_ng_p*
aac(3)IV_F aac(3)IV_R	ATCGACTGATGTCATCAGCG CGAGCTGAAGAAAGACAAT	Amplification of *aac(3)IV*
gusA_ver_F gusA_ver_R	GGCGGCTACACGCCCTTCGA TGATGGGCCGGGTGGGGTC	Amplification of *gusA* internal fragment

* *Restriction sites are underlined in primer sequence*.

#### Construction of the *dbv3*, *dbv4*, and *nocRI* Overexpression Plasmids

The coding sequences of *dbv3* (2,635 bp) and *dbv4* (1,006 bp) were amplified from the A40Y cosmid (Table 1; Marcone et al., 2010a) using Q5 High-Fidelity DNA Polymerase and the dbv3_F/R or dbv4_F/R primer pairs (Table 2). The obtained amplicons were digested with *Eco*RI and *Eco*RV and cloned into pSET152A cleaved with the same enzymes. The resulting plasmids were named pSAD3 and pSAD4. To generate a vector for the overexpression of both *dbv3* and *dbv4*, the regions containing the coding sequences of both genes were amplified using the dbv4_F/dbv3_R primer pair and cloned into pSET152A in a similar fashion, generating pSAD3–4.

To construct the vector for overexpression of *nocRI* (the *dbv3* ortholog from *N. coxensis*), the coding sequence of *A3G7_RS0138355* was amplified from the genomic DNA of *N. coxensis* isolated using the Kirby procedure (Kieser et al., 2000) using Q5 High-Fidelity DNA Polymerase and the orfR1_F/R primer pair (Table 2). The obtained amplicon (2,661 bp) was digested with *Eco*RI and *Eco*RV and cloned into pSET152A cut with the same enzymes to generate pSAR1. All of the generated recombinant plasmids were verified by restriction endonuclease mapping and sequencing at BMR Genomics (University of Padua, Italy).

### Conjugative Transfer of Plasmids Into *Nonomuraea* spp. and Verification of the Recombinant Strains

Conjugative transfer of plasmids into *N. gerenzanensis* was performed essentially as described previously (Marcone et al., 2010c). All recombinant plasmids were transferred individually into the non-methylating *E. coli* ET12567 pUZ8002 and the resulting derivatives used as donor strains for intergeneric conjugation. To prepare fresh vegetative mycelium of *N. gerenzanensis* prior to conjugal transfer, one vial of WCB was inoculated into 50 ml of VSP medium (250 ml Erlenmeyer flask with 10 ø5 mm glass beads) and incubated for 48 h on the orbital shaker at 220 rpm and at 30°C. The mycelium was collected by centrifugation (10 min, 3,220 × *g*), washed twice with sterile 20% v/v glycerol, resuspended in the same solution to a final volume of 20 ml, and stored at −80°C. 1 ml of mycelial suspension was mixed with approximately 10^9^ of donor *E. coli* cells and the mixtures were plated on well dried VM0.1 agar plates supplemented with 20 mM of MgCl_2_. After 12–16 h of incubation at 30°C, each plate was overlaid with 1 ml of sterile deionized water containing 1.25 mg of apramycin-sulfate and 750 μg of nalidixic acid sodium salt. Transconjugants were selected as resistant to 50 μg/ml of apramycin-sulfate.

Spore suspensions of *N. coxensis* were prepared from lawns grown on ISP3 agar for 7 days. Spores from one plate were collected in deionized water and filtered through one layer of Miracloth (Merck KGaA, Darmstadt, Germany) to remove vegetative mycelial fragments. Then, spores were pelleted from a 50 ml suspension by centrifugation (15 min, 3,220 × *g*), resuspended in 1 ml of 15% v/v glycerol, and stored at −80°C. For conjugation, approx. 10^6^ spores were mixed with 10^7^ *E. coli* donor cells and plated on VM0.1 agar plates supplemented with 20 mM of MgCl_2_. The overlay for the selection of transconjugants was performed as described previously for *N. gerenzanensis*.

To verify the integration of promoter-probe vectors, an ~1 kbp internal fragment of *gusA* was amplified from the genomic DNA of recombinant *N. gerenzanensis* or *N. coxensis* strains using the gusA_ver_F/R primer pair (Table 2). To verify the integration of pSET152A, *aac(3)IV* was amplified using the aac(3)IV_F/R primer pair (Table 2). To verify the integration of pSAD4, an ~1 kbp fragment of pSAD4 was amplified with the PAM_seq_F/dbv4_R primer pair (Table 2), in which PAM_seq_F anneals within the *aac(3)IVp* sequence. Verification of pSAD3 and pSAD3–4 integration was made by amplification of an ~2 kbp fragment (for pSAD3) or an ~3 kbp fragment (for pSAD3–4), using the PAM_seq_F/dbv3_seq_R primer pair (Table 2), in which dbv3_seq_R anneals in the middle of *dbv3.* Finally, to verify the integration of pSAR1 an ~2 kbp fragment was amplified using the PAM_seq_F/orfR1mid_EcoRV_R (Table 2) primer pair, in which orfR1mid_EcoRV_R anneals in the middle of *nocRI*.

### β-Glucuronidase Activity Assay

β-Glucuronidase (GusA) activity in *Nonomuraea* strains growing on VM0.1 agar medium was assessed by adding, after 6 days of cultivation at 30°C, 10 μl drops of 5-bromo-4-chloro-3- indolyl-β-D-glucuronide (X-Gluc, Thermo Fisher Scientific, Waltham, MA, United States) 50 mg/ml in DMSO to the surfaces of the lawns. Chromogenic conversion of X-Gluc into the blue-colored 5,5′-dibromo-4,4′-dichloro-indigo was monitored after 1 h of incubation. For the quantitative measurements of GusA activity, *Nonomuraea* strains were grown in liquid media. One WCB vial of each of the strains was inoculated into a baffled 500 ml Erlenmeyer flask containing 100 ml of E26 (*N. gerenzanensis* strains) or of VSP (*N. coxensis* strains). After 72 h of cultivation, 10% v/v of this preculture was transferred into a baffled 500 ml Erlenmeyer flask containing 100 ml of FM2 (*N. gerenzanensis* strains) or ISP2 (*N. coxensis* strains). To induce *P21-cmt*-driven *gusA*-expression, cumate was added at the final concentration of 50 μM to cultures carrying pGCymRP21 24 h after inoculation. After 120 h of cultivation, mycelial lysates were prepared as previously reported by Horbal et al., 2013. Glucuronidase activity was measured as previously described (Myronovskyi et al., 2011; Horbal et al., 2013) using a spectrophotometric assay following the conversion of the colorless *p*-nitrophenyl-β-D-glucuronide (Thermo Fisher Scientific, Waltham, MA, United States) into the colored *p*-nitrophenol at 415 nm using an Infinite 200 PRO microplate reader (Tecan, Switzerland). Glucuronidase activity was normalized to dry biomass weight as previously reported (Marcone et al., 2014). One unit of activity is defined as the amount of enzyme that is able to convert 1 μM of substrate in 1 min.

### A40926 Production

One WCB vial was inoculated into 300 ml baffled flasks containing 50 ml of vegetative medium E26 with 10 glass beads (ø5 mm). Flask cultures were incubated for 72 h on a rotary shaker at 220 rpm and 30°C and then used to inoculate (10% v/v) 500 ml baffled Erlenmeyer flasks containing 100 ml of FM2 medium or a 3-l P-100 Applikon glass reactor (height 250 mm, ø130 mm) equipped with a AD1030 Biocontroller and AD1032 motor, and containing 2 l of the same production medium. Cultivations in FM2 in shake-flasks were conducted at 30°C and 220 rpm. Bioreactor fermentations were conducted at 30°C, with stirring at 450 rpm (corresponding to 1.17 m/s of tip speed) and 2 l/min aeration rate. Dissolved oxygen (measured as % pO2) was monitored using an Ingold polarographic oxygen electrode. The pH values of culture broths were monitored using a pH meter. Foam production was controlled by adding Hodag antifoam (Hodag Chemical Corporation, Chicago, IL, United States) through an antifoam sensor. Samples were collected at regular cultivation time intervals and analyzed to estimate biomass (dry weight), glucose consumption (Diastix sticks, Bayer AG, Leverkusen, Germany), and A40926 production.

### HPLC Analysis of Culture Extracts

A40926 was extracted from *Nonomuraea* spp. cultures as previously reported (Marcone et al., 2014). Chromatography was performed with a VWR Hitachi diode array L-2455 HPLC system with detection at 254 nm. The A40926 titers in the batch cultivations were estimated by injecting 50 μl of sample onto a 5 μm-particle-size Ultrasphere ODS (Beckman) HPLC column (4.6 by 250 mm) and eluting at a flow rate of 1 ml/min with a 30 min linear gradient from 15 to 64% of phase B. Phase A was 32 mM HCOONH_4_ (pH 7) – CH_3_CN [90:10 (vol/vol)], and phase B was 32 mM HCOONH_4_ (pH 7) – CH_3_CN [30:70 (vol/vol)]. A volume of 50 μl of a pure sample of 200 μg/ml A40926 (Sigma-Aldrich, St. Louis, MO, United States) was used as an internal standard.

### Tools for the Bioinformatics Analysis

Blastp was used to search for homologs (Altschul, 1990); protein sequence alignments were performed with Clustal Omega (EMBL-EBI, Sievers et al., 2011).

## Results

### Genetic Manipulation of *Nonomuraea coxensis*, a Novel Putative Producer of a A40926-Like Molecule

According to the 16S rRNA gene-based reconstruction of *Nonomuraea* phylogeny (Dalmastri et al., 2016), *N. coxensis* occupies a relatively distant position from *N. gerenzanensis*. Conversely, mining the partially sequenced genome of *N*. *coxensis* (ASM37988v1), we found a close homolog of the *N. gerenzanensis* regulatory gene *dbv3* (locus *A3G7_RS0138355* in the *N. coxensis* genome, coding for WP_020547054.1 with 86.6% predicted amino acid sequence identity with Dbv3, ESM Figure 1). Dbv3 is a unique LuxR-regulator controlling the expression of the A40926 BGC and it is not closely related to the better-characterized family of Tei16*-like regulators controlling teicoplanin biosynthesis, sharing only 32% of amino acid sequence identity with Tei16* (Yushchuk et al., 2019). This *dbv3*-like gene (we named it *nocRI*) was found on a short *N. coxensis* contig (NZ_KB904006) flanking a gene coding for a putative StrR-like transcriptional regulator apparently orthologous to *dbv4* (locus *A3G7_*RS0138355, coding for WP_026215141.1 with 94.39% of amino acid sequence identity with Dbv4); *dbv4* is the other known cluster-situated regulatory gene in the *dbv* BGC and we named the *N. coxensis* homolog *nocRII* (ESM Figure 2). AntiSMASH (Blin et al., 2019) analysis of *N. coxensis* genome revealed the presence of three short contigs (NZ_KB904006, NZ_KB903995, NZ_KB903969, ESM Figure 3) covering the majority of a *dbv-*like BGC, suggesting that *N. coxensis* might produce A40926 or a very similar GPA. Thus, we considered *N. coxensis* an interesting candidate for developing *Nonomuraea*-targeted genetic tools.

**Figure 1:**
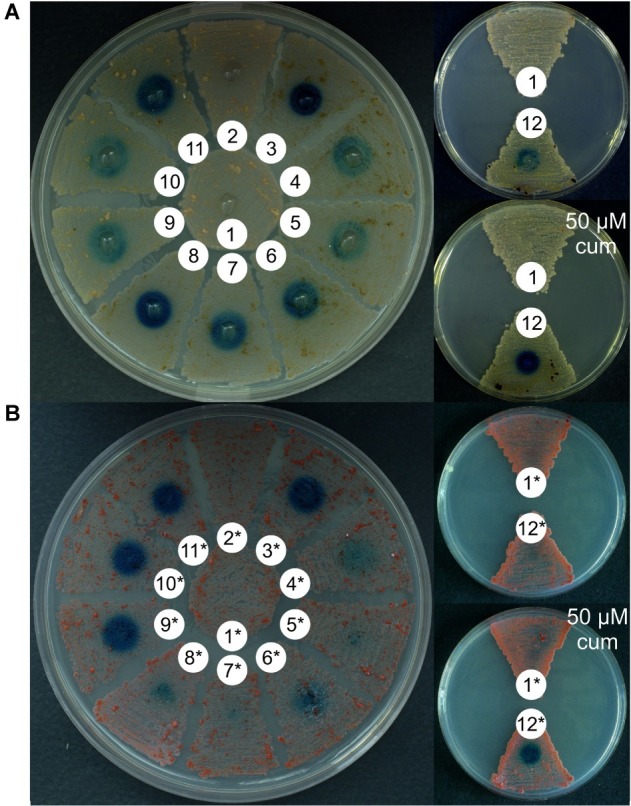
Comparison of GusA activity in recombinant strains of *N. gerenzanensis* **(A)** and *N. coxensis* **(B)**, carrying the following promoter-probe vectors: 2, 2* – pTEGA (*gusA* under the control of *ermEp*); 3, 3* – pSAGA (*gusA* under the control of *aac(3)IVp*); 4, 4* – pHBP (*gusA* under the control of *hrdB_ng_p*); 5, 5* – pRBP2 (*gusA* under the control of *rpoB_Rng_p*); 6, 6* – pRBP1 (*gusA* under the control of *rpoB_ng_p*); 7, 7* – pSBP (*gusA* under the control of *ssb_ng_p*); 8, 8* – pRLP (*gusA* under the control of *rpsL_ng_p*); 9, 9* – pGUSmoeE5script (*gusA* under the control of *moeE5p*); 10, 10* – pGT2p (*gusA* under the control of *tei2p*); 11, 11* – pGBP (*gusA* under the control of *gyrB_ng_p*); 12, 12* – pGCymRP21 (*gusA* under the control of *P21-cmt*). The control parental strains (1, *N. gerenzanensis* and 1*, *N. coxensis*) do not display chromogenic conversion of X-Gluc. Strains were cultivated for 6 days on VM0.1 medium.

**Figure 2:**
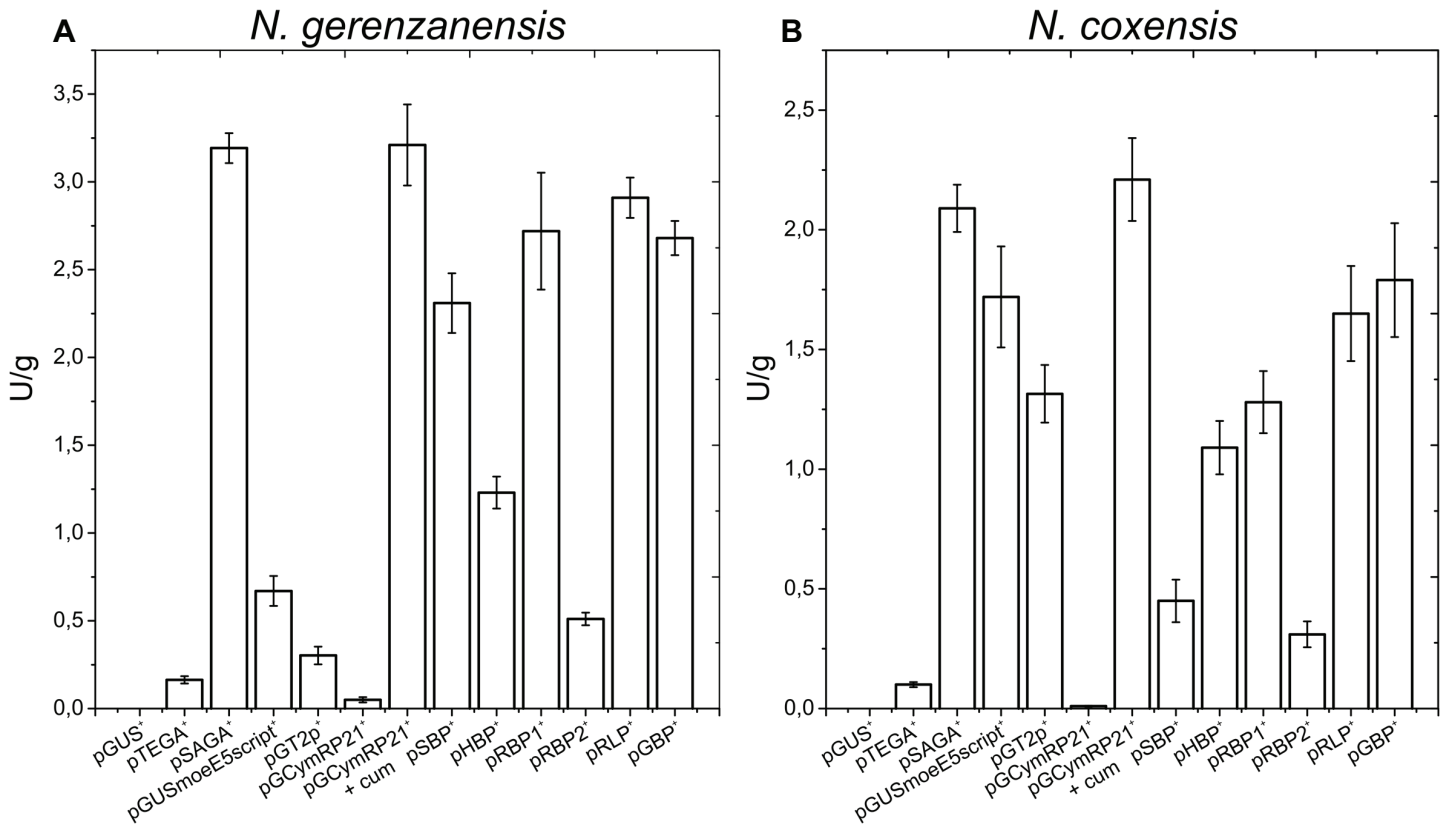
Quantitative measurement of GusA activity (U/gram of dry biomass) in *N. gerenzanensis* **(A)** and *N. coxensis* **(B)** recombinant strains grown in liquid media for 120 h (see growth curves in ESM Figure 5), carrying different promoter-probe plasmids. Control strains carried the promoterless pGUS vector and exhibited no GusA activity. Activities are the mean values of three independent experiments. Error bars represent standard deviations.

**Figure 3:**
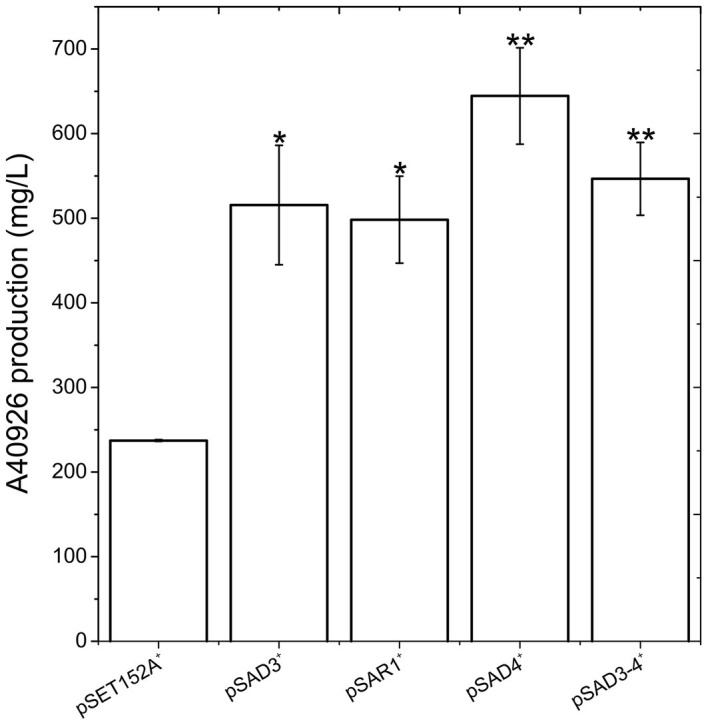
A40926 production in *N. gerenzanensis* carrying the empty vector pSET152A^+^ and in its recombinant strains overexpressing *dbv3* (pSAD3), *nocRI* (pSAR1), *dbv4* (pSAD4), and co-expressing *dbv3* and *dbv4* genes (pSAD3–4) cultivated in FM2 industrial medium at 500 ml Erlenmeyer flasks-scale. A40926 production was measured after 120 h of cultivation. The results given represent three independent fermentations, error bars represent standard deviations. Statistical significance of the differences in A40926 production between the control and the recombinant strains was estimated using Welch’s *t*-test: ^*^p < 0.05; ^**^p < 0.01.

Initially, we tried to transfer *φC31*-based integrative plasmids (Table 1) into *N. coxensis* by using the protocol of intergeneric conjugation optimized for conjugal transfer from a DNA-non-methylating *E. coli* donor strain to *N. gerenzanensis* vegetative mycelium (Marcone et al., 2010c). Transconjugants were obtained at a very low frequency (*ca.* 1 × 10^−7^) and only for the relatively small pSAGA-based promoter-probe vectors (approx. 6 kbp). Increasing the amount of donor and recipient cells, changing the time of overlay, and adjusting medium composition (including increasing or decreasing MgCl_2_ concentration) did not allow the transfer of the larger pGUS-based promoter-probe vectors (such as pGUSmoeE5script or pGCymRP21, both more than 9 kbp). Since, unlike *N. gerenzanensis*, *N. coxensis* sporulates abundantly when grown on ISP3 agar medium (ESM Figure 4), we tried to use spores for conjugal transfer. This resulted in transfer rates of approximately 1 × 10^−3^ when 10^6^ spores were mixed with 10^7^ *E. coli* donor cells, regardless of plasmid size.

**Figure 4:**
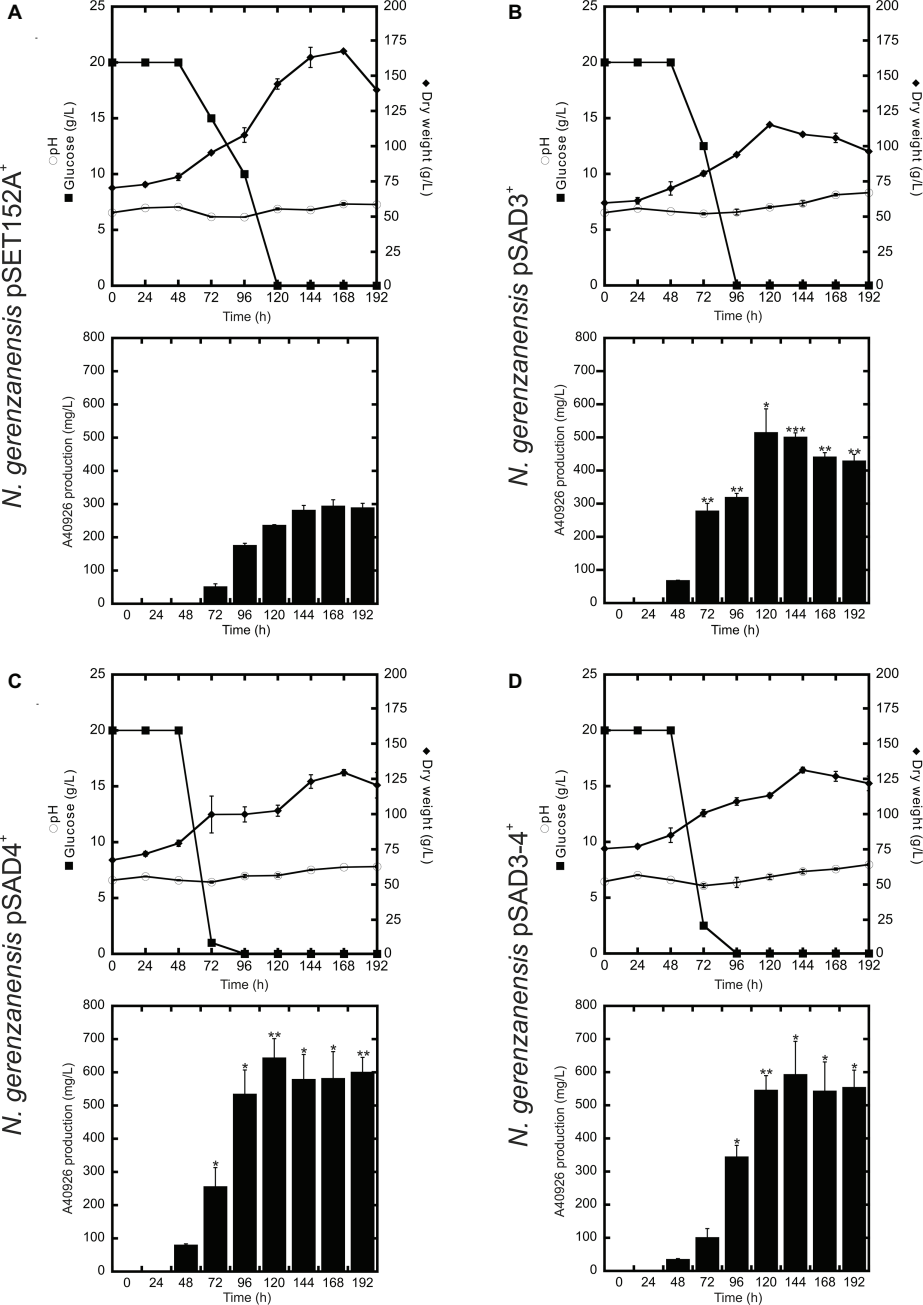
Time courses of *N. gerenzanensis* pSET152A^+^ **(A)** and recombinant strains overexpressing *dbv3* **(B)**, *dbv4* **(C)** and co-expressing both genes **(D)** cultivated in FM2 industrial medium in 500 ml Erlenmeyer flasks. Glucose consumption (filled rectangles), biomass accumulation (filled rhombi), pH (circles), and A40926 production were monitored every 24 h. Results given are mean values of three independent experiments. Error bars represent standard deviations. Statistical significance of the differences in A40926 production between the control and the recombinant strains was estimated using Welch’s *t*-test: ^*^p < 0.05; ^**^p < 0.01; ^***^p < 0.001.

### Using the GusA-Reporter System to Assess the Activity of Native and Heterologous Promoters in *Nonomuraea* Species

The set of *φC31*-based integrative plasmids transferred to *N. gerenzanensis* and *N. coxensis* were promoter-probe vectors utilizing the GusA reporter system and carrying a selection of native and heterologous promoters, the latter having been used previously to drive gene expression in streptomycetes or actinoplanetes (Horbal et al., 2013, 2014a; Makitrynskyy et al., 2013). Glucuronidase activity of the recombinant *Nonomuraea* strains was assessed qualitatively (on agar plates) and quantitatively (in cell lysates obtained from mycelium grown in liquid medium). Both of the *N. coxensis* and *N. gerenzanensis* wild type strains did not display any glucuronidase activity (Figure 1). The heterologous promoters tested were: *aac(3)IVp* (in pSAGA) – the apramycin acetyltransferase gene promoter, derived from pSET152A (Horbal et al., 2013); *ermEp* (in pTEGA, Yushchuk et al., 2020a) – the erythromycin resistance gene promoter from pTES (Herrmann et al., 2012); *moeE5p* (in pGUSmoeE5script) – the *S. ghanaensis* moenomycin biosynthesis gene *moeE5* promoter (Makitrynskyy et al., 2013); *tei2p* (pGT2p, Yushchuk et al., 2020b) – the *A. teichomyceticus* teicoplanin resistance gene *tei2* promoter; and *P21*, a synthetic promoter fused with the cumate inducible *Pseudomonas putida* F1 *cmt* operon operator (in pGCymRP21, Horbal et al., 2014a). In parallel, we tested the activity of six native promoters derived from *N. gerenzanensis* house-keeping genes: *gyrB_ng_p* (in pGBP – the promoter of the DNA gyrase B subunit gene); *hrdB_ng_p* (in pHBP – the promoter of the RNA polymerase σ-factor RpoD gene); *ssb_ng_p* (in pSBP – the promoter of the single-stranded DNA-binding protein); *rpsL_ng_p* (in pRLP – the promoter of the SSU ribosomal protein S12p gene); *rpoB_Rng_p* (in pRBP2 – the promoter of the rifamycin-resistant RNA polymerase subunit β gene); and *rpoB_ng_p* (in pRBP1 – the promoter of the rifamycin-sensitive RNA polymerase subunit β gene). In contrast to the wild type strains, all of the recombinant derivatives grown on VM0.1 agar plates converted X-Gluc to its colored derivative 5,5′-dibromo-4,4′-dichloro-indigo, albeit to different extents (Figure 1). *N. gerenzanensis* pSAGA^+^ and *N. coxensis* pSAGA^+^, carrying *aac(3)IVp*, produced the most intensive color (Figures 1A,B), while the chromogenic conversion of X-Gluc in *N. gerenzanensis* pTEGA^+^ and *N. coxensis* pTEGA^+^, carrying the *ermEp*, was only slightly visible. In strains carrying the inducible pGCymRP21, GusA activity was induced by the addition of 50 μM of cumate, proving inducible gene expression (Figures 1A,B). However, in *N. gerenzanensis* pGCymRP21^+^, a basal level of GusA activity was detected even in the absence of cumate (Figure 1A). Contrary to this, no basal level of expression from *P21-cmt* was detected in *N. coxensis* pGCymRP21^+^ (Figure 1B).

When the glucuronidase activity present in cell lysates obtained from liquid cultures grown for 120 h (late exponential/early stationary growth phase, see ESM Figure 5) was measured using a spectrophotometric assay, overall these quantitative results (normalized for the dry weight of the differently growing strains) correlated with those observed on agar plates: *aac(3)IVp* behaved as a strong promoter in both *N. gerenzanensis* and *N. coxensis*, surpassed only by *cmt-P21p* when induced by the addition of 50 μM cumate (Figures 2A,B). Also *rpsL_ng_p* and *hrdB_ng_p* proved to be strong promoters in both of the *Nonomuraea* spp. whereas the weakest was the *ermEp* (Figures 2A,B). Interestingly, the activity of *rpoB_ng_P* was higher than that of *rpoB_Rng_p* (Figures 1, 2), consistent with previously reported data about differences in transcription levels of the two alleles (Vigliotta et al., 2004).

**Figure 5:**
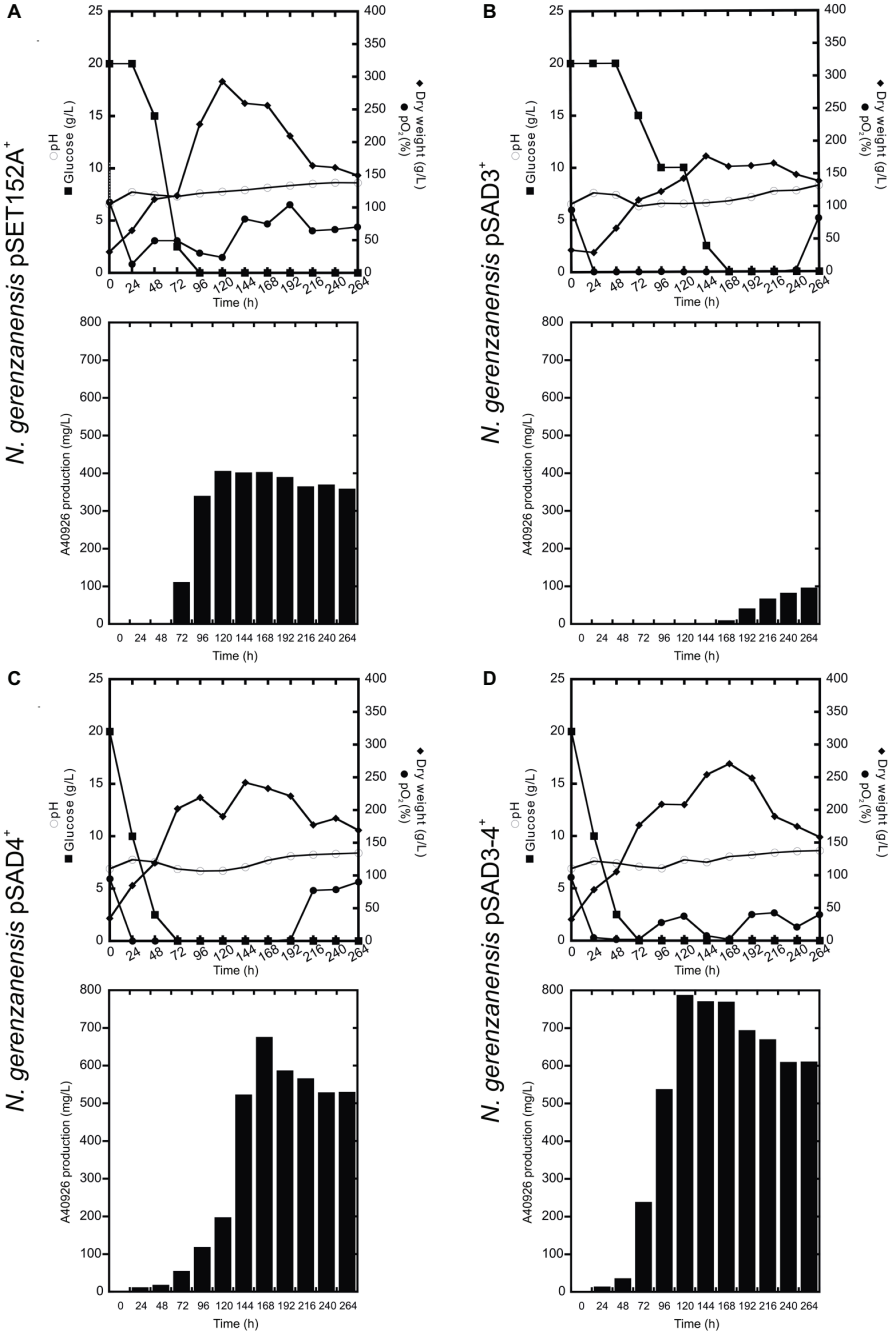
Time courses of *N. gerenzanensis* pSET152A^+^ **(A)** and recombinant strains, overexpressing *dbv3* **(B)**, *dbv4* **(C)** and co-expressing both genes **(D)** cultivated in 2 l FM2 industrial medium in a 3 l fermenter. Glucose consumption (filled rectangles), biomass accumulation (filled rhombi), pH (circles), O_2_ level (filled circles), and A40926 production were monitored every 24 h.

### Knowledge-Based Generation of A40926 Overproducing Strains

Since *aac(3)IVp* was identified as the strongest constitutive promoter for *Nonomuraea* spp., we used it to overexpress *dbv3* and *dbv4* in *N. gerenzanensis*. Both genes were cloned into the integrative pSET152A vector (Horbal et al., 2013) yielding the recombinant vectors pSAD3 and pSAD4, respectively (Table 1). Benefiting from the neighboring positions of *dbv3* and *dbv4*, we also cloned them together, generating pSAD3–4, where *dbv4* was directly under the control of *aac(3)IVp*, but *dbv3* remained under the control of its native promoter (Table 1). Additionally, the *dbv3-*like *nocRI* from *N. coxensis* was cloned into pSET152A, generating pSAR1, and transferred to *N. gerenzanensis* to determine if it could improve A40926 production, and to assess possible cross-talk between the regulators from the two different *Nonomuraea* spp. All the *N. gerenzanensis* recombinant strains were grown for 120 h in parallel with the control strain carrying the empty vector and A40926 production was measured (Figure 3). The production of A40926 in *N. gerenzanensis* carrying pSET152A reached almost 250 mg/l after 120 h of cultivation (Figure 3). The four recombinant strains overexpressing the cluster-situated regulatory genes produced more antibiotic than the control strain (Figure 3). A40926 production in *N. gerenzanensis* pSAD3^+^ and pSAR1^+^ was comparable (*ca*. 500 mg/l), proving that *nocRI* had a similar impact in *N. gerenzanensis* as *dbv3.* The recombinant strain carrying pSAD3–4 vector produced slightly more, around 550 mg/l, whereas the best producer in these conditions was the strain overexpressing *dbv4*, which produced more than 650 mg/l.

### Time Courses of A40926 Production in *Nonomuraea gerenzanensis* Recombinant Strains at Flask and at Bioreactor Scale

Although further investigations will be devoted to the expression of the heterologous *nocRI* and *nocRII in N. gerenzanensis*, in our strain improvement work we then focused on *N. gerenzanensis* strains overexpressing native regulators. Consequently, *N. gerenzanensis* strains containing pSET152A, pSAD3, pSAD4, or pSAD3–4 were grown for 192 h in parallel with the control strain carrying the empty vector using the previously optimized industrial medium FM2 (Marcone et al., 2010a, 2014) at flask scale. Samples for the analysis of dry weight, pH, glucose consumption, and A40926 production were collected at regular 24 h intervals. All three recombinant strains expressing the regulatory genes from *aac(3)IVp* accumulated detectable amounts of A40926 earlier than the empty vector control (Figure 4). A40926 production reached its peak after 120 h of growth for *N. gerenzanensis* pSAD3^+^ (*ca.* 500 mg/l) (Figure 4B) and pSAD4^+^ (nearly 650 mg/l) (Figure 4C), whereas in pSAD3–4^+^ the maximum productivity (*ca*. 600 mg/l) was delayed to 144 h (Figure 4D). The control strain produced *ca.* 300 mg/l after 144–168 h from inoculation (Figure 4A). Although glucose consumption was faster in the recombinant strains containing the cloned regulatory genes in comparison to the control, they accumulated less biomass than *N. gerenzanensis* pSET152A^+^ (Figure 4). Maximum biomass production was around 125 g/l (dry weight) in the overexpression strains versus the 175 g/l produced by the empty vector control strain. These data suggest that part of the consumed glucose was used by the strains carrying the regulatory genes under the control of the strong constitutive promoter *aac(3)IVp* to support antibiotic production at the detriment of biomass formation.

When the cultivation of recombinant strains was scaled up in 3 l vessel-bioreactors containing 2 l working volume of FM2, all of the strains produced significantly more A40926 and grew better than in flask culture with the exception of *N. gerenzanensis* pSAD3^+^ (Figure 5). The control strain with the empty vector grew and produced the antibiotic faster than at the flask level (Figure 5A). Maximum biomass (300 g/l dry weight, more than the double of that achieved in flasks) and A40926 production (nearly 400 mg/l) were reached after 120 h from inoculation; glucose was completely consumed within 96 h versus the 120 h needed at flask level. The recombinant strain pSAD4^+^ grew more (maximum biomass production of 220 g/l after 144 h of growth) than in flask culture, although less than the control strain in the bioreactor. Glucose was consumed faster than in the control strain and glucose concentration tended to zero at 48 h of fermentation (Figure 5C), and antibiotic production reached a peak of 700 mg/l after 168 h. The best performance in terms of A40926 productivity in the bioreactor was shown by the pSAD3–4^+^ strain, which grew better (nearly 250 g/l dry weight biomass) than in flask culture and produced the maximum concentration of the antibiotic (800 mg/l) after 168 h of cultivation (Figure 5D). Conversely, the pSAD3^+^ strain showed a reduced biomass production in comparison to all of the other strains, with consumption of glucose markedly delayed and A40926 production starting very late (after 168 h of cultivation) and never exceeding 100 mg/l (Figure 5B). Microscopical observation of pSAD3^+^ strain showed that the mycelium was highly fragmented (data not shown), suggesting some kind of physiological stress resulting from *dbv3* overexpression. Some fragmentation of the pSAD3^+^ strain (as well as of the pSAR1 strain carrying the *dbv3-*like *nocRI* from *N. coxensis*) was also observed in flask culture, in contrast to the other strains which produced dense mycelial pellets; the fragmentation was less pronounced, indicating that scaling up in the bioreactor dramatically enhanced this effect. Interestingly, in the best performer at the bioreactor scale – the pSAD3–4^+^ strain – *dbv4* was expressed from the strong constitutive *aac(3)IVp* but *dbv3* was left under its endogenous promoter, suggesting that the balance between the expression level of the two cluster-situated regulatory genes is important for optimal improvement of A40926 production.

## Discussion

Apart from some reports on cultivating and manipulating the industrially valuable A40926 producer *N. gerenzanensis* (Stinchi et al., 2003, 2006; Marcone et al., 2010a,b,c; Alt et al., 2019) and the kistamicin producer *Nonomuraea* sp. ATCC 55076 (Greule et al., 2019), we are not aware of any other attempt to develop genetic tools for manipulating *Nonomuraea* spp. However, some other glycopeptide producers, like *A. teichomyceticus*, already possess well-developed toolkits for genetic manipulation which has greatly simplified investigations in these strains (Horbal et al., 2013; Yushchuk et al., 2016). Therefore, our first goal in this work was to develop a set of genetic tools for manipulating diverse species of *Nonomuraea*. To do this, we decided to work in parallel with the better-known A40926 producer, recently re-classified as *N. gerenzanensis* (Dalmastri et al., 2016), and with the little investigated *N. coxensis*, which was isolated in Bangladesh in 2007 (Ara et al., 2007). Although the available *N. coxensis* genome sequence is still incomplete, we could identify three contigs covering most of a *dbv*-like gene cluster including the *dbv*-like cluster-situated regulatory genes, which we named *nocRI* (*dbv3* homolog) and *nocRII* (*dbv4* homolog). The next step of our work will be additional sequencing to yield a properly annotated *N. coxensis* genome. Interestingly, during the course of our investigations, Waglechner et al. (2019), systematically screening the available sequences in genomic databases, also reported the presence of a BGC encoding for a A40926-like GPA in the genome of *N. coxensis*. In addition, the same authors reported that a newly isolated *Nonomuraea* sp. WAC01424 possess a BGC which could produce another A40926-related compound (Waglechner et al., 2019). Our preliminary analysis of this BGC suggests that it could be a sulfated A40926-like GPA, lacking the aliphatic side chains.

In the meantime, we tested both in *N. gerenzanensis* and *N. coxensis*, a set of heterologous and native promoters (the latter derived from a set of house-keeping genes in *N. gerenzanensis*) with the final goal of using them for driving gene expression in these strains. Besides the practical outcome of this screening (all of the generated promoter-probe vectors could be easily used as expression vectors simply by exchanging *gusA* for a gene of interest, offering a set of variable tools for gene expression), it is interesting to observe that the studied promoters had similar strengths in the two phylogenetically distant *Nonomuraea* species and that the native promoters from *N. gerenzanensis* worked similarly in *N. coxensis*. Consistently, we detected a comparable difference in the two strains between the strength of promoters driving the expression of rifamycin-sensitive and rifamycin-resistant *rpoB* alleles of *N. gerenzanensis* (*rpoB_ng_p* and *rpoB_Rng_p*). Since we found the two *rpoB* alleles in *N. coxensis* draft genome (on a short genomic scaffolds KB904038 and KB904038), we might suppose that their manipulation could improve antibiotic production in this strain as already reported for *N. gerenzanensis* (Vigliotta et al., 2004). More generally, these vectors should be useful for the genetic manipulation of other members of the genus *Nonomuraea*, which have the potential to produce novel valuable specialized metabolites (Sungthong and Nakaew, 2015; Nazari et al., 2017).

As in the case of *Actinoplanes* spp. (Horbal et al., 2013), the strongest heterologous promoter was *aac(3)IVp*, although some *N. gerenzanensis* native promoters like *hrdB_ng_p* and *rpsL_ng_p* appeared to have comparable strength and merit further investigations. When we used *aac(3)IVp* to overexpress the cluster-situated regulatory genes *dbv3* and *dbv4* from the *N. gerenzanensis dbv* gene cluster and *nocRI* (*dbv3*-like) from the A40926-like BGC of *N. coxensis*, the recombinant *N. gerenzanensis* strains produced significantly more A40926 than the parental strain. The evidence that the heterologous expression of *nocRI* increased A40926 production in *N. gerenzanensis* confirmed its role in regulating the expression of a A40926-like BGC in *N. coxensis*. Additionally, it represents another case of cross-talk between regulators controlling GPA BGCs in producing actinomycetes (Spohn et al., 2014). Our next goal will be to investigate if and how (in which cultivation conditions) *N. coxensis* produces A40926 or a A40926-like molecule.

Previous work (Lo Grasso et al., 2015) reported that overexpression of *dbv3* (under the control of the thiostrepton-inducible tipA* promoter in the integrative plasmid pIJ8600) in *N. gerenzanensis* increased A40926 production from 13 to 27 mg/l using the laboratory medium R3 (Lo Grasso et al., 2015). In this paper, we tested the real industrial potential of overexpressing not only *dbv3*, but also *dbv4*, and *dbv3* and *dbv4* together, cloning them under the strong constitutive *aac(3)IVp* promoter, scaling up their cultivation at bioreactor scale and using a previously optimized industrial medium where A40926 is produced in hundreds of milligrams per liter (Marcone et al., 2010a, 2014). At the bioreactor level, where strains could have a different performance from the flask-cultivation due to different mixing and mass transfer rates of nutrients and oxygen, the best performer was the strain carrying both *dbv4* under *aac(3)IVp* and *dbv3* under its own endogenous promoter. This strain produced nearly 800 mg/l of A40926, which is twice that of the parental strain grown under the same conditions. Conversely, the strain carrying only *dbv3* expressed from *aac(3)IVp* showed reduced production capacity and an altered phenotype particularly after scaling up from flask to bioreactor; in contrast, the *dbv4* overexpressing recombinant grew similarly under both conditions. It is widely recognized that any potentially higher producing mutant or derivative needs to be validated in a bioreactor-scale fermentation since unpredictable discrepancies in strain performance can occur during scaling up from flask culture (Lee and Kim, 2015). In the case of the *dbv3* overexpressing recombinant, we believe that the fragmented mycelial phenotype, which was much more apparent in the bioreactor, could be a specific consequence of the overexpression of the *dbv7* gene encoding a d,d-carboxypeptidase known as VanYn (Binda et al., 2012). The level of VanYn activity in cell extracts from the *dbv3*-carrying recombinant cultivated at bioreactor scale was found to be much higher than in the parental strain (unpublished data). Consistent with this, overexpression of VanYn altered the mycelial morphology in *N. gerenzanensis* as well as in heterologous hosts such as streptomycetes strains (Binda et al., 2013).

In conclusion, only a few GPAs are used in clinical practice and those produced by semi-synthesis from natural products, such as dalbavancin derived from A40926, are still quite expensive. Dalbavancin is the first antibiotic designated as a Qualified Infection Diseases Product by the FDA because of its potency, extended dosing interval, and unique dose regimen, but its cost largely exceeds that of first-generation GPAs and consequently its use in hospitals is still limited (Chiasson and White, 2016; Agarwal et al., 2018). Improving A40926-producing strains might lead to a decrease in the cost of dalbavancin. As demonstrated in this paper, A40926 production could be significantly enhanced by manipulating the expression of *dbv* cluster-situated regulators. An important and often-neglected aspect is testing the genetic stability and productivity of the selected recombinant strains in a fully developed industrial process at bioreactor level, which mimics the conditions of antibiotic large scale production. We were able to demonstrate here that the improvements we made to A40926 production levels in shake flasks were also achieved in the bioreactor, indicating the relevance of this approach to industrial-scale strain improvement.

## Data Availability Statement

All datasets generated for this study are included in the article/Supplementary Material.

## Author Contributions

OY, EB, MB, and FM conceived and designed the experiments and wrote the paper. OY, AA-V, GM, and EB performed the experiments. OY, GM, and EB analyzed the data.

### Conflict of Interest

The authors declare that the research was conducted in the absence of any commercial or financial relationships that could be construed as a potential conflict of interest.
