# Supplementary material for: New Molecular Tools for Regulation and Improvement of A40926 Glycopeptide Antibiotic Production in Nonomuraea gerenzanensis ATCC 39727
Source: Front Microbiol. 2020 Jan 21;11:8. doi: 10.3389/fmicb.2020.00008 (PMC6985074; doi:10.3389/fmicb.2020.00008)
Supplement: Supplementary file 1 [file Data_Sheet_1.PDF]

Supplementary Material

1 Supplementary Data

Compositions of media used in the work:

Unless otherwise stated, components are from Sigma-Aldrich, St. Louis, MO, United States.

ISP2 (g/l of distilled water):
Yeast extract – 4;
Malt extract – 10;
Dextrose – 4;
Agar – 20;
pH 7.5.
ISP3 (g/l of tap water):
Fine ground whole oats (Kozub, Poltava, Ukraine) – 34;
Agar – 20;
pH 7.5.
VSP (g/l of distilled water):
Soluble starch (Difco, Franklin Lakes, NJ, United States) – 24;
Dextrose – 1;
Meat extract – 3;
Yeast extract – 5;
Tryptose – 5;
L-proline – 0.5;
Sucrose – 50;
pH 7.5.
VM0.1 (g/l of distilled water):
Soluble starch (Difco) – 2.4;
Dextrose – 0.1;
Meat extract – 0.3;
Yeast extract – 0.5;
Tryptose – 0.5;
Agar – 20;
pH 7.2.
E26 (g/l of distilled water):
Dextrose – 25;
Soy flour – 20;
Yeast extract – 4;
NaCl – 1.25;
$CaCO_3$ – 5;

pH 7.5.
FM2 (g/l of distilled water):
Dextrose – 30;
Soy flour – 30;
Yeast extract – 8;
Malt extract – 15;
$CaCO_3$ – 5;
L-valine – 1;
pH 7.5.
SFM (g/l of distilled water):
Mannitol – 20;
Soy flour – 20;
Agar – 20;
pH 7.5.

2 Supplementary Figures and Tables

2.1 Supplementary Figures

```
NocRI           LTNRLGDERVLFGRDRELKSLTELLDSTAAGRGGMAVIRGPLVGGKTAVLHELGMRSIAA 60
Dbv3            ---------VLFGRDRELKSLTRLLDSTAAGRGGVAVITGPVVGGKTAILHELGMRSIAA 51
                         *************.***********:*** **:******:***********

NocRI           GVRLVRAGCTPAERSLDWGVADQILGRGAAERLTAHRDGDAVEEVCDSLFQMAEANPVLL 120
Dbv3            GIRLVTARCTPAEQSLDWGVADQILGRGAAERLTARRGGDAVEDVCVSLFQMAEANPILL 111
                *:*** * *****:********************:*.*****:** **********:**

NocRI           AIDDVDLADDPSLQAILAMAPQLANTRMMIAVTICPDRPPARLLDVAGTLLRLPGVQLVE 180
Dbv3            TIDDVDLADDPSLLAILSMTPLLTDTRMMIAVTICQDRPPAPLPHVAESLLRLPGIELVE 171
                :************ ***:*:* *::********** ***** * .** :******::***

NocRI           LPLLPRPAVRRFAAEHLGAETADQIADDLYRFSGGSPLLVRALIEDQEAGAPGLAVGDSF 240
Dbv3            LPLLPRPAVRQFATEHLGAETADQLADDLYRFSGGSPLLVRALIEDQEAGAPGLVVGDSF 231
                **********:**:**********:*****************************.*****

NocRI           MSAVATCVHGFEPEAVRVAEAVAVLGEHATPDAVGELVGIAPSAAMRSMGMLARAGLLAR 300
Dbv3            MSAVAACVHGCEPEAVRVAEAVAVLGEHATPDAVGELVGIAPPAATRSMGMLERAGLLAG 291
                *****:**** ******************************* ** ****** ******

NocRI           GRFRHEAGGRAVLGRMTSYGRMDLLRRAAEIVYRRGGPLPAVATHLLEAGWSGEEWAYDV 360
Dbv3            GRFRHEAGRLAVLGRMTSYGRMEILRRAAEILHRRGGPPSAVATRLLEAGWSGEEWAFDV 351
                ********  ************::*******::*****  ****:************:**

NocRI           LVDAGRQAFREGDFVAVMKCLRLALASGWGRPGRLDVKVMLAAAEWRVDPAVAARHLPDL 420
Dbv3            LVEAGRQAFDEGDFVAVMKCLRLALASGWGTPRRLDVKVMLAAAEWRVDPAVAARHVPDL 411
                **:****** ******************** * ***********************:***

NocRI           LDAARSGALRGSHGAELFRQFLWYGRFADAGELIDRLRPAVADRDADVSLIGMCHVHPAL 480
Dbv3            LDATRSGALRGSHGMELFRQLLWYGRFADAAELIDRLRPSVADRDADASLIAMCHVHPVL 471
                ***:********** *****:*********.********:*******.***.******.*

NocRI           LDRLPRSARGSTGHTIEDARRILHQAEPTDEAMDSIISALMALLLGGVPDVATSCETLLK 540
Dbv3            LDRLPRSARGSMGQTVEDARRILRQAEPTDEAMDSIISALMALLLGGVSEVAASCETLLK 531
                *********** *:*:*******:************************ :**:*******

NocRI           EPRVTKAPTWKAIISAVQAEAAWRKGDLAGAEAHAREALTILQPSGWGVAIGAPLSTLLH 600
Dbv3            EPGVTKAPTWKAIISAIRAETAWRKGDLAGAEAHAQEALTILQPSGWGVAIGAPLSTLLH 591
                ** *************::**:**************:************************

NocRI           AQTAMGHLDDAKATVDVPMPRETAETAFGIGYELARAHYHLATDQPRIAFAGFQACGQAI 660
Dbv3            AQTAMGHLDEAKATVAVPMPRETAETAFGIGYELARAHYHLVTEQPRAAFAGFLACGQAV 651
                *********:***** *************************.*:*** ***** *****:

NocRI           QRWGCSLSCVFPWRLGAAQACLQLGWRRRAADLVTAQILDTAPDDLRTYGIALRLLAQLS 720
Dbv3            QRWGSSLSDVVPWRLGAARACLQLGWRRRAADLVTAQIAHTSSGDLRTYGVALRLHAQLS 711
                ****.*** *.*******:******************* .*: .******:**** ****

NocRI           KPGQRRQLLMESVNALETAQDRYQLALALSDVAGNFQLKGGKHEARAYWVRAQELARECN 780
Dbv3            KPAQRQRLLMQSVDALEAAQDRYQLALSLCDLAGTPQLKGGKDEARAYWVRAQELARECN 771
                **.**::***:**:***:*********:*.*:**. ******.*****************

NocRI           AKPLMRRLAAEHDHAEAAPLSGAERRVAVLAARGHTNREIAEALYITRSTVEQHLTRIYR 840
Dbv3            AKPLMRRLAAQHDHGETAPLSGAERRVAVLAARGHTNREIAEALYITRSTVEQHLTRIYR 831
                **********:***.*:*******************************************

NocRI           KLNIQTRGDLSDLFAAYIAEEATTTAGRTA-------         870
Dbv3            KLHVQTRGDLGNLFAADIADKATATAGREPREAVRL*         867
                **::******.:**** **::**:****
```

Supplementary Figure 1. Alignment of the amino acid sequences of Dbv3 and its orthologue from *Nonomuraea coxensis* – NocRI. Alignment was generated using ClustalOmega (EMBL-EBI).

```
NocRII      VDPTGVDIVALPVVEIELSRLSSVYSPRTSGEDPEHVETLLSAQGELPPILVHRPTMRVI        60
Dbv4        VDPTGVDIATLPVVEIELSRLSSVYSPRTSGEDPEHVETLLSAQGELPPILVHRPTMRVI        60
            ********.:**************************************************

NocRII      DGLHRLRVARVRGETKIAVRLIDGTESDAFVLAVEANVRHGLPLSLADRKRAAVQIIGTH        120
Dbv4        DGLHRLKVARVRGETTISVRLIDGTESDAFVLAVEANVRHGLPLSLADRKRAAVRIIGTH        120
            ******:********.*:************************************:*****

NocRII      PQWSDRRVASATGISAGTVADLRKRRGQDGDEARIGRDGRIRPVDSSEGRRLAAEIIRSH        180
Dbv4        PQWSDRRVASATGISAGTVADLRRRRGQGGDEARIGRDGRIRPVDSSEGRRLAAELIRSH        180
            ***********************:****.**************************:****

NocRII      PDLSLRQVAKQVGISPETVRDVRGRLEQGESPIPDGSRRLRAKPESLRRPEQDFGHAGGR        240
Dbv4        PDLSLRQVAKQVGISPETVRDVRGRLEHGESPIPDGSRRLRTKPELLRRAEQDFGHVDGR        240
            ***************************:*************:*** *** ******..**

NocRII      DRQAVLERLKADPALRLTETGRILLRMLSLHSIDGQEWERILRGVPPHWDAVVARCARDH        300
Dbv4        DRQAVLERLKADPALRLTETGRILLRMLSLHSIDGQEWERILRGVPPHWGTVVARCARDH        300
            *************************************************.:*********

NocRII      AQIWAAFADRLEGRATDLAAG       321
Dbv4        AQIWAAFADRLEGRATDLAAG       321
            *********************
```

Supplementary Figure 2. Alignment of the amino acid sequences of Dbv4 and its orthologue from *N. coxensis* – NocRII. Alignment was generated using ClustalOmega (EMBL-EBI).

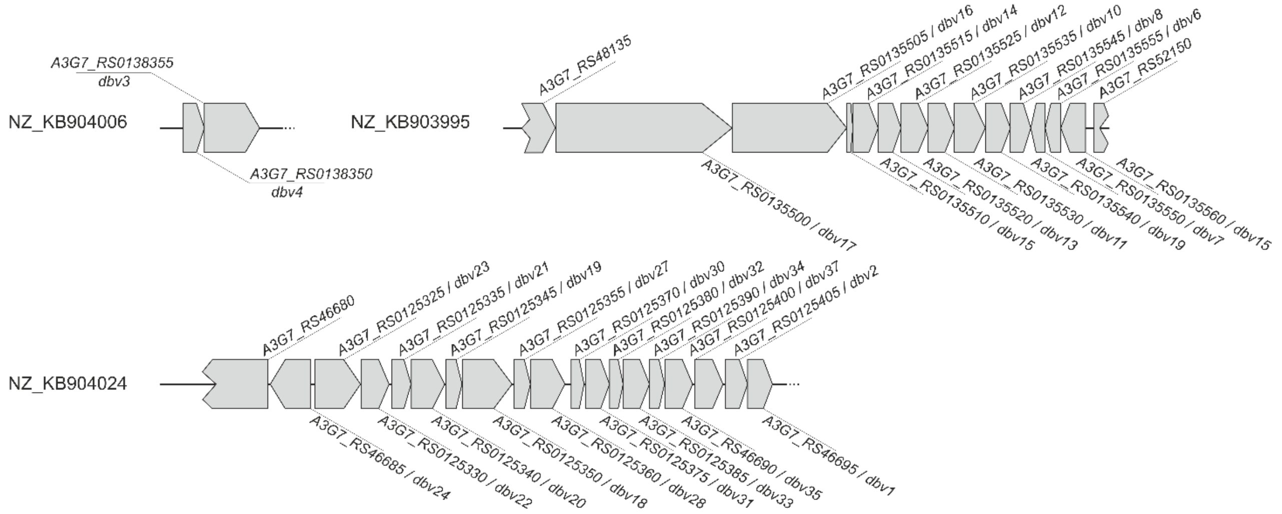

Supplementary Figure 3. Scheme of the parts of putative GPA BGC from *N. coxensis* that could be found on three contigs available in GenBank. Locus tags for individual genes are given together with the names of putative orthologues from *dbv* cluster.

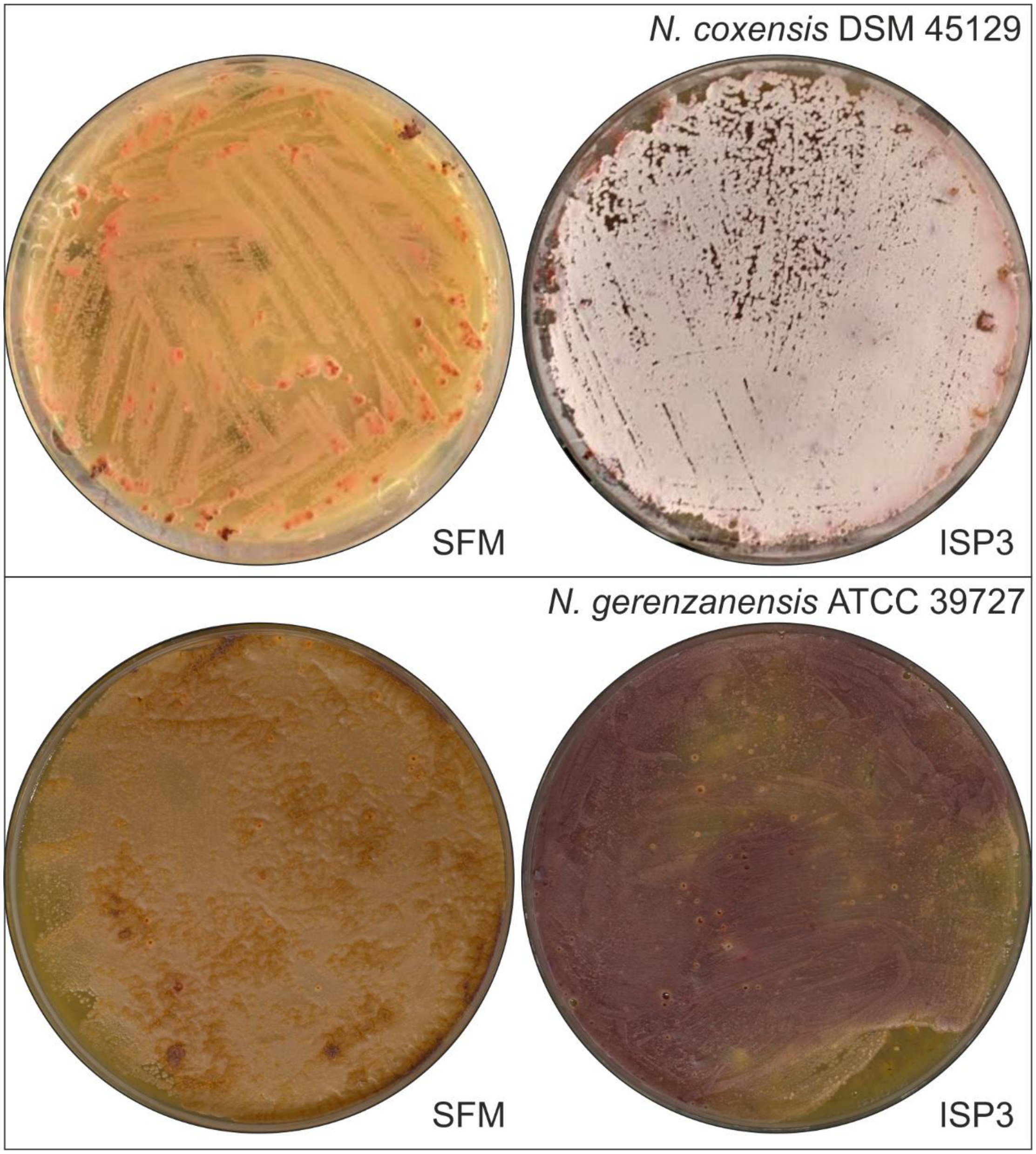

Supplementary Figure 4. 7-day-growth of *N. coxensis* DSM 45129 and *Nonomuraea gerenzanensis* ATCC 39727 on SFM and ISP3 media. Both strains did not differentiate spores on SFM, which indeed promotes sporulation of *Streptomyces* spp. and of other uncommon actinobacteria like *Actinoplanes* spp. (Kieser et al., 2000; Gren et al., 2016). Only *N. coxensis* produces abundant spores on ISP3 in these conditions. *N. gerenzanensis* was reported to produce very few spores only after 14-21 days of cultivation on ISP3 (Dalmastri et al., 2016).

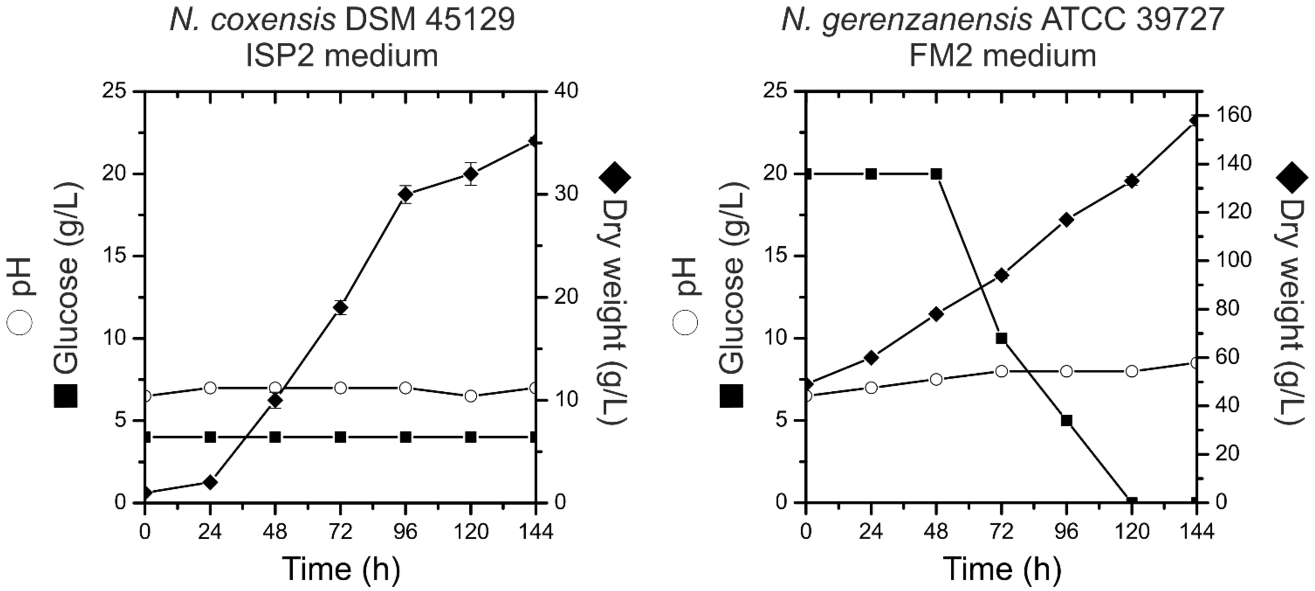

Supplementary Figure 5. Growth curves of *N. coxensis* DSM 45129 and *N. gerenzanensis* ATCC 39727 in ISP2 and FM2 liquid media, respectively. Glucose consumption (rectangles), biomass accumulation (rhombi) and pH (circles) were monitored every 24 h. Results given are mean values of three independent experiments. Error bars represent standard deviations.

3 Supplementary references

Dalmastri, C., Gastaldo, L., Marcone, G. L., Binda, E., Congiu, T., and Marinelli, F. (2016). Classification of *Nonomuraea* sp. ATCC 39727, an actinomycete that produces the glycopeptide antibiotic A40926, as *Nonomuraea gerenzanensis* sp. nov. *International Journal of Systematic and Evolutionary Microbiology* 66, 912–921. doi:10.1099/ijsem.0.000810.

Gren T., Ortseifen. V., Wibberg, D., Schneiker-Bekel, S., Bednarz, H., Niehaus, K., Zemke, T., Persicke, M., Pühler, A., Kalinowski, J., (2016). Genetic engineering in *Actinoplanes* sp. SE50/110 – development of an intergeneric conjugation system for the introduction of actinophage-based integrative vectors. *Journal of Biotechnology* 232, 79-88. doi: 10.1016/j.jbiotec.2016.05.012.

Kieser, T., Bibb, M. J., Buttner, M. J., Chater, K. F., and Hopwood, D. A. (2000). *Practical Streptomyces Genetics.* Norwich: John Innes Foundation.
